# Brain Functional Changes in Stroke Following Rehabilitation Using Brain-Computer Interface-Assisted Motor Imagery With and Without tDCS: A Pilot Study

**DOI:** 10.3389/fnhum.2021.692304

**Published:** 2021-07-16

**Authors:** Mengjiao Hu, Hsiao-Ju Cheng, Fang Ji, Joanna Su Xian Chong, Zhongkang Lu, Weimin Huang, Kai Keng Ang, Kok Soon Phua, Kai-Hsiang Chuang, Xudong Jiang, Effie Chew, Cuntai Guan, Juan Helen Zhou

**Affiliations:** ^1^NTU Institute for Health Technologies, Interdisciplinary Graduate Programme, Nanyang Technological University, Singapore, Singapore; ^2^Center for Sleep and Cognition, Center for Translational MR Research, Yong Loo Lin School of Medicine, National University of Singapore, Singapore, Singapore; ^3^Department of Biomedical Engineering, Faculty of Engineering, National University of Singapore, Singapore, Singapore; ^4^Institute for Infocomm Research, Agency for Science Technology and Research, Singapore, Singapore; ^5^School of Computer Science and Engineering, Nanyang Technological University, Singapore, Singapore; ^6^Singapore Bioimaging Consortium, Agency for Science Technology and Research, Singapore, Singapore; ^7^Queensland Brain Institute and Centre for Advanced Imaging, The University of Queensland, Brisbane, QLD, Australia; ^8^School of Electrical and Electronic Engineering, Nanyang Technological University, Singapore, Singapore; ^9^Division of Neurology, University Medicine Cluster, National University Health System, Singapore, Singapore; ^10^Department of Electrical and Computer Engineering, National University of Singapore, Singapore, Singapore; ^11^Integrative Sciences and Engineering Programme (ISEP), National University of Singapore, Singapore, Singapore

**Keywords:** functional magnetic resonance imaging, stroke, amplitude of low-frequency fluctuation, regional homogeneity, functional connectivity, brain-computer interface-assisted motor imagery, transcranial direct current stimulation

## Abstract

Brain-computer interface-assisted motor imagery (MI-BCI) or transcranial direct current stimulation (tDCS) has been proven effective in post-stroke motor function enhancement, yet whether the combination of MI-BCI and tDCS may further benefit the rehabilitation of motor functions remains unknown. This study investigated brain functional activity and connectivity changes after a 2 week MI-BCI and tDCS combined intervention in 19 chronic subcortical stroke patients. Patients were randomized into MI-BCI with tDCS group and MI-BCI only group who underwent 10 sessions of 20 min real or sham tDCS followed by 1 h MI-BCI training with robotic feedback. We derived amplitude of low-frequency fluctuation (ALFF), regional homogeneity (ReHo), and functional connectivity (FC) from resting-state functional magnetic resonance imaging (fMRI) data pre- and post-intervention. At baseline, stroke patients had lower ALFF in the ipsilesional somatomotor network (SMN), lower ReHo in the contralesional insula, and higher ALFF/Reho in the bilateral posterior default mode network (DMN) compared to age-matched healthy controls. After the intervention, the MI-BCI only group showed increased ALFF in contralesional SMN and decreased ALFF/Reho in the posterior DMN. In contrast, no post-intervention changes were detected in the MI-BCI + tDCS group. Furthermore, higher increases in ALFF/ReHo/FC measures were related to better motor function recovery (measured by the Fugl-Meyer Assessment scores) in the MI-BCI group while the opposite association was detected in the MI-BCI + tDCS group. Taken together, our findings suggest that brain functional re-normalization and network-specific compensation were found in the MI-BCI only group but not in the MI-BCI + tDCS group although both groups gained significant motor function improvement post-intervention with no group difference. MI-BCI and tDCS may exert differential or even opposing impact on brain functional reorganization during post-stroke motor rehabilitation; therefore, the integration of the two strategies requires further refinement to improve efficacy and effectiveness.

## Introduction

Robot-assisted motor imagery (MI) and brain-computer interface (BCI) system is an advanced technique for improving motor function recovery in post-stroke rehabilitation. Motor imagery allows the imagination of movements without physical execution, and electroencephalography-based brain-computer interfaces (EEG-BCIs) enables interaction with the environment using brain signals (Dodakian et al., 2014; Bockbrader et al., 2018). By combining robot-assisted MI and BCI, the brain-computer interface-assisted motor imagery (MI-BCI) system enables stroke patients to move the impaired limb driven by a robotic arm via neural signals from the motor imagery of the arm and provides multisensory feedback to patients to enhance motor learning and recovery (Ang et al., 2014, 2009; Ang and Guan, 2015; Wang H. et al., 2019; Foong et al., 2020).

Transcranial direct current stimulation (tDCS) is another promising technique for stroke rehabilitation. tDCS is a non-invasive brain stimulation technique that uses constant, low direct current delivered via electrodes (anode and cathode) on the head to provide a reversible and focal way for neuroplasticity modulation (Nitsche and Paulus, 2001, 2000). tDCS has been shown to modulate activity in both the motor and visual cortices and more recently has been shown to directly influence the excitability of the spinal cord (Zaghi et al., 2010; Butler et al., 2013; Elsner et al., 2018). Furthermore, tDCS has been reported to have a long-term effect lasting beyond the time of stimulation which might indicate the ability to improve motor function through network reorganization and modulation of brain plasticity (Reidler et al., 2011; Allman et al., 2016; Kashiwagi et al., 2018; Liu et al., 2018). Although the mechanism of tDCS has not been fully revealed yet, converging evidence demonstrated the efficacy of tDCS for motor function improvements in stroke (Allman et al., 2016; Butler et al., 2013; Elsner et al., 2018; Hordacre et al., 2018; Lindenberg et al., 2010). Given the benefits in motor function recovery from both MI-BCI and tDCS, it is unclear whether the combination of MI-BCI and tDCS would further enhance functional motor recovery in post-stroke rehabilitation. The underlying neural mechanisms of combined MI-BCI and tDCS are also largely unknown.

Resting-state functional magnetic resonance imaging (rs-fMRI) provides an indirect measure of neuronal activity and connectivity based on regional blood-oxygen-level-dependent (BOLD) signal fluctuations without any imposed task (Biswal et al., 1995). Various analytic methods have been proposed to derive complementary measures of brain functional dynamics from rs-fMRI data (Lv et al., 2018). Amplitude of Low-Frequency Fluctuation (ALFF) measures the total power of the BOLD signal within the low-frequency range between 0.01 and 0.1 Hz, which represents local brain activity (Zang et al., 2007). Regional Homogeneity (ReHo) is a voxel-based measure of the similarity between the time-series of a given voxel and its nearest neighbors, which represents local connectivity as calculated by the Kendall coefficient of concordance of the BOLD time-series (Zang et al., 2004). Seed-based functional connectivity (FC) approach measures the temporal correlations between the activity in a seed region and BOLD signals across the brain (Biswal et al., 1995). Canonical functional networks such as the default mode network (DMN) and the executive control network (ECN) have been consistently derived from FC mapping in rs-fMRI (Ng et al., 2016; Chong et al., 2019, 2017a; Cheng et al., 2021). These measurements provide important insights into the brain functional dynamics, disease mechanisms, and brain-behavior associations (Wang et al., 2016a; Chong et al., 2017b; Puig et al., 2018; Zhao et al., 2018; Chen et al., 2019; Zhang et al., 2020). The combination of different measurements incorporates both regional and global information and thus complements each other to build an efficient model for brain analysis.

Nevertheless, little work has been done to examine the brain functional changes in stroke after MI-BCI and tDCS-based intervention. Most of the previous work focused on cross-sectional brain ALFF/ReHo/FC changes in stroke patients in comparison with healthy controls. In terms of regional activity, some studies reported lower ALFF in the motor cortex and lower ALFF/ReHo in the default mode network (DMN) in stroke patients compared to healthy controls (Tsai et al., 2014; Zhu et al., 2015; La et al., 2016) while others reported no differences or contradictory results in the DMN (Liu et al., 2015). On the other hand, disrupted FC in the motor cortex and DMN were widely reported in stroke patients with motor function impairment and cognitive impairment (Park et al., 2011, 2014; Golestani et al., 2013; Tuladhar et al., 2013; Ding et al., 2014; De Bruyn et al., 2018; Puig et al., 2018; Lee et al., 2019). Among the limited work on brain functional changes post-intervention in stroke, emerging evidence demonstrated enhancement of FC in the motor and sensory-related networks after motor imagery training (Sun et al., 2013; Wang et al., 2018). To our knowledge, no study has directly compared the brain functional changes between stroke rehabilitation using MI-BCI and tDCS and how these brain measures relate to clinical outcomes such as motor recovery.

In this study, we examined the alterations in brain functional activity and connectivity in chronic subcortical stroke patients using ALFF, ReHo, and FC. The changes in the neural function of stroke patients pre- and post-intervention were investigated in the MI-BCI only group and MI-BCI combined with tDCS group separately. We hypothesized that 1) lowered ALFF/ReHo and disrupted FC in the motor cortex and DMN would be observed in stroke patients compared to healthy controls at baseline; 2) stroke patients would have higher ALFF/ReHo/FC in the motor cortex and DMN post-intervention, the degree of which would differ between the MI-BCI and the MI-BCI + tDCS groups. Lastly, we investigated whether these alterations in ALFF/ReHo/FC post-intervention would correlate with motor function recovery measured by Fugl-Meyer Assessment (FMA) scores.

## Materials and Methods

### Participants

Forty-two stroke patients aged from 21 to 70 years with moderate to severe motor impairments and 11 age-matched healthy controls were recruited in this study. The inclusion criteria for stroke patients were (1) first-ever subcortical stroke > 9 months before the study enrollment; and (2) unilateral moderate to severe upper extremity motor impairment measured by the upper extremity component of the FMA (score range 11–45). The exclusion criteria for stroke patients were (1) epilepsy, neglect, cognitive impairment, and other neurological or psychiatric diseases; (2) severe arm pain; (3) severe spasticity measured by the Modified Ashworth Scale in the shoulder or elbow (score > 2); (4) contraindications to tDCS; (5) weak grip strength measured by a dynamometer (weight < 10 kg); and (6) participation in other interventions or trials that target post-stroke motor recovery. Twenty-three stroke patients were excluded because they either declined to participate in the study or failed to meet the inclusion or BCI performance criteria. Detailed demographics of the remaining 19 patients and 11 healthy controls who completed the training and scans were provided in Table 1. This study was conducted in accordance with the Code of Ethics of the World Medical Association and approved by the National Healthcare Group Domain-Specific Review Board. It was also registered on ClinicalTrials.gov (Clinical Trial Registration Unique Identifier: NCT01897025, date of registration: July 8, 2013; https://clinicaltrials.gov/ct2/show/NCT01897025). Written inform consent was obtained from all participants.

**TABLE 1:** Subject demographics.

	HC (*N* = 11)	Stroke patients		
		Total (*N* = 19)	MI-BCI + tDCS (*N* = 10)	MI-BCI (*N* = 8^a^)
Age (years)	56.7 ± 4.5	54.1 ± 10.6	52.1 ± 11.7	54.6 ± 8.5
Gender (M/F)	6/5	14/5	6/4	8/0
Handedness (R/L)	11/0	17/2	9/1	7/1
Affected hemisphere (R/L)	–	11/8	5/5	5/3
Stroke type (I/H)	–	13/6	6/4	6/2
Stroke location (C/S)	–	1/18	1/9	0/8
Stroke onset to therapy (days)	–	1037 ± 598	1052 ± 721	1076 ± 466
BCI screening performance (%)	–	76.5 ± 10.4	79.1 ± 9.4	73.3 ± 10.3

*^a^One subject was involved in baseline comparisons but excluded from intervention analysis as the second scan did not pass quality control.*

*No significant difference was found between any groups for all variables listed here. M, Male; F, Female; R, Right; L, Left; I, Infarction; H, Hemorrhage; C, Cortical; S, Subcortical; HC, Healthy Controls.*

### MI-BCI and tDCS Study Design

Nineteen stroke patients were randomized into the MI-BCI + tDCS group (*n* = 10) and MI-BCI group (*n* = 9) with matching pre-training FMA scores. The MI-BCI + tDCS group received 20-min real tDCS, in which direct current was applied using a saline-soaked pair of surface sponge electrodes at an intensity of 1 mA with the anode placed over the M1 motor cortex of the ipsilesional hemisphere and the cathode placed over the contralesional M1 while the MI-BCI group received 20-min sham tDCS, in which current ramped up and down to give subjects the sensation of the stimulation. Both groups completed 10 sessions of MI-BCI training within 2 weeks. Each MI-BCI training was 40 min and consisted of 160 trials. In each trial, subjects were prepared with a visual cue and instructed to perform motor imagery by an instruction cue. The EEG segment of 0.5– 4.5 s from the instruction cue was then extracted to classify the EEG segment to perform online detection of MI or the idle condition using the Filter Bank Common Spatial Pattern (FBCSP) algorithm (Ang et al., 2012). The MIT-Manus robot provided movement feedback if the motor imagery was detected. More details of the study design can be found in Supplementary Materials and Ang and Guan (2015).

Motor function was assessed by the upper extremity component of the FMA, which is a stroke-specific, performance-based impairment index to assess motor functioning, balance, sensation and joint functioning in patients with post-stroke hemiplegia (Fugl-Meyer et al., 1975). Motor function assessments were conducted (1) 1 week before training, (2) immediately after training, and (3) 4 weeks after training.

### Image Acquisition

All stroke patients completed three MRI scans: at screening, 1 week before training, and 4 weeks after training. Healthy controls underwent two MRI scans: at screening and 3 weeks after screening. The MRI scans conducted 1 week before training for patients and 3 weeks after screening for healthy controls were considered as baseline. For patients, the MRI scans conducted 1 week before training and 4 weeks after training were regarded as pre- and post-intervention respectively.

MRI data were collected using a 3T scanner (TIM Trio, Siemens, Germany) with a 32-channel head array coil. The scan protocol included a T1-weighted magnetization prepared rapid gradient-echo (MPRAGE) sequence (inversion time = 900 ms, repetition time = 1,900 ms, echo time = 2.5 ms, flip angle = 9°, field of view = 256 × 256 mm^2^, 176 sagittal slices, matrix size = 256 × 256, and voxel size = 1 mm^3^ isotropic), and a resting state T2^∗^-weighted echo planar imaging (repetition time = 1,750 ms, echo time = 30 ms, flip angle = 74°, field of view = 220 × 220 mm^2^, voxel size = 3.4 mm^3^ isotropic, slice thickness = 3.4 mm, 33 axial slices). During the 7-min rs-fMRI, participants were instructed to remain relaxed and awake with their eyes closed and not engage in any specific activity.

### Image Analyses

#### fMRI Preprocessing

Preprocessing of fMRI images was performed using FMRIB software library (FSL) and analysis of functional neuroimaging (AFNI) following our standard protocol (Cox, 1996; Ng et al., 2016; Wang et al., 2016b; Chong et al., 2017a). The preprocessing steps consist of: (i) removal of first six volumes for magnetic field stabilization; (ii) slice-time correction; (iii) motion correction, (iv) despiking; (v) spatial smoothing (6-mm FWHM Gaussian kernel); (vi) grand mean scaling; (vii) band-pass temporal filtering (0.009 – 0.1 Hz); (viii) detrending (removal of first and second order); (ix) use of Boundary-Based Registration (BBR) for co-registration of T1 image (Greve and Fischl, 2009) and non-linear registration tool (FNIRT) for subsequent registration to the Montreal Neurological Institute (MNI) 152 space. Specifically, the left hemisphere was defined as the ipsilesional hemisphere and all images with lesions at the right hemisphere were flipped to investigate the effect of lesion location. Additionally, regression of nuisance signals (white matter, cerebrospinal fluid (CSF), whole-brain global signals, and six motion parameters) and motion scrubbing were performed for analysis of FC to avoid spurious connectivity (Power et al., 2012; Power et al., 2014).

Quality of co-registration and normalization were visually inspected. One post-intervention image with movement more than 4 mm was discarded. Thus after quality control, data of 19 stroke patients and 11 healthy control remained at baseline. Data of 10 subjects in the MI-BCI + tDCS group and 8 subjects in the MI-BCI group remained for pre- and post-intervention analysis.

#### Derivation of the Voxelwise Amplitude of Low Frequency Fluctuation Maps

The ALFF was calculated using a toolbox for Data Processing & Analysis for Brain Imaging (DPABI) (Yan et al., 2016). For each voxel, a discrete Fourier transform was performed on the resting-state time series. The ALFF was computed by measuring the average square root of the total power spectrum between 0.01 and 0.10 Hz on a voxel-by-voxel basis.

#### Derivation of Voxelwise Regional Homogeneity Maps

The ReHo maps were also produced using DPABI toolbox. ReHo was calculated in a voxel-wise manner using Kendall’s concordance coefficient (KCC) across the time series of a centered cubic cluster of 27 voxels. A large ReHo value for a given voxel indicates a high local synchronization of rs-fMRI signal among neighboring voxels and vice versa.

#### Derivation of Default Mode and Somatomotor Network Functional Connectivity

FC analysis was performed for two networks, default mode network and somatomotor network, which were previously reported to show changes in ALFF and ReHo analysis in our study. DMN and SMN were defined using Region of Interest (ROI) from a FC-based 114-node parcellation (Buckner et al., 2011). 24 ROIs were included for DMN and 10 ROIs were included for SMN (ROI names and peak coordinates in Supplementary Table 1). BOLD time series of each ROI were extracted for every subject and Pearson’s correlation between the mean time series of every pair of ROIs were computed. An FC matrix was produced for every subject by Fisher’s r-to-z transformation of correlation coefficients. FC within the DMN and SMN, as well as between the DMN and SMN were consequently derived by averaging the corresponding cells in the matrix.

### Statistical Analysis

Baseline differences in ALFF, ReHo, and FC between stroke patients and healthy controls were examined using two-sample *t*-tests. Voxelwise analyses (i.e., ReHo and ALFF) were thresholded using a height threshold of *p* < 0.01 (uncorrected) and extent threshold of *p* < 0.05 (familywise-error (FWE) corrected).

To examine the effects of intervention as well as interaction effects between intervention and group on FMA scores, ALFF and ReHo maps, we conducted mixed-effect Analyses of Variance (ANOVA) with intervention as within-subjects factor and group as between-subject factor. *Post hoc* tests were subsequently performed to examine intervention effects in each group separately. Voxelwise analyses (i.e., ReHo and ALFF) were thresholded using a height threshold of *p* < 0.01 (uncorrected) and extent threshold of *p* < 0.05 (FWE corrected).

Finally, associations between functional activity/connectivity and clinical outcomes (motor function improvement measured by FMA scores) were examined using Pearson’s correlation. Eight-mm spheres created in MARSBAR (Brett et al., 2002) with peak coordinates extracted from the above interaction effect maps were selected as ROIs for ALFF and ReHo. ALFF/ReHo changes in the ROIs were correlated with changes in FMA. FC changes within DMN/SMN and between DMN and SMN were associated with changes in FMA.

## Results

### Improved Motor Function After Stroke Rehabilitation

**FIGURE 1:**
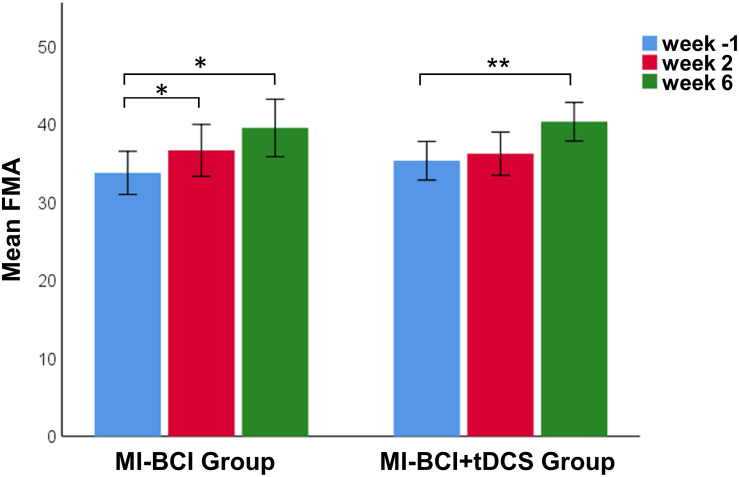
Motor recovery after MI-BCI intervention with and without tDCS. FMA scores at baseline and post intervention. Significant FMA gains comparing to baseline were only found at 4 weeks after training for both MI-BCI and MI-BCI + tDCS group (MI-BCI group: 5.8 ± 6.0, *p* = 0.030; MI-BCI + tDCS group: 5.0 ± 4.4, *p* = 0.006) but not immediately post training (MI-BCI group: 2.9 ± 4.2, *p* = 0.096; MI-BCI + tDCS group: 0.9 ± 3.0, *p* = 0.362). No significant intergroup differences were observed at any time point during the study. **p < 0.01; *p < 0.05. Error bars indicate ± 1SE.

There was no difference between the MI-BCI group and MI-BCI + tDCS group at baseline in terms of age, post-stroke time, or FMA scores (Table 1). A 3 (intervention: baseline, immediately after training, 4 weeks after training) × 2 (group: MI-BCI, MI-BCI + tDCS) mixed effects ANOVA performed on FMA scores indicated significant intervention effect (*p* = 0.002) but no interaction effect between intervention and group (*p* = 0.450), suggesting that both groups gained significant motor function improvement post-intervention with no group difference observed either immediately or 4 weeks after training. *Post hoc* tests further showed that significant FMA gains compared to baseline were only found at 4 weeks after training (MI-BCI group: 5.8 ± 6.0, *p* = 0.030, effect size = 0.62; MI-BCI + tDCS group: 5.0 ± 4.4, *p* = 0.006, effect size = 0.64) but not immediately post-training (MI-BCI group: 2.9 ± 4.2,*p* = 0.096, effect size = 0.32; MI-BCI + tDCS group: 0.9 ± 3.0, *p* = 0.362, effect size = 0.11) (Figure 1). Furthermore, the MI-BCI group showed greater motor function improvement than the MI-BCI + tDCS group at week 2, although this was not statistically significant (MI-BCI group: 2.9 ± 4.2 vs. MI-BCI + tDCS group: 0.9 ± 3.0, *p* = 0.130, effect size = 0.55).

### Abnormal ALFF/ReHo in the SMN and DMN in Stroke Patients at Baseline

**FIGURE 2:**
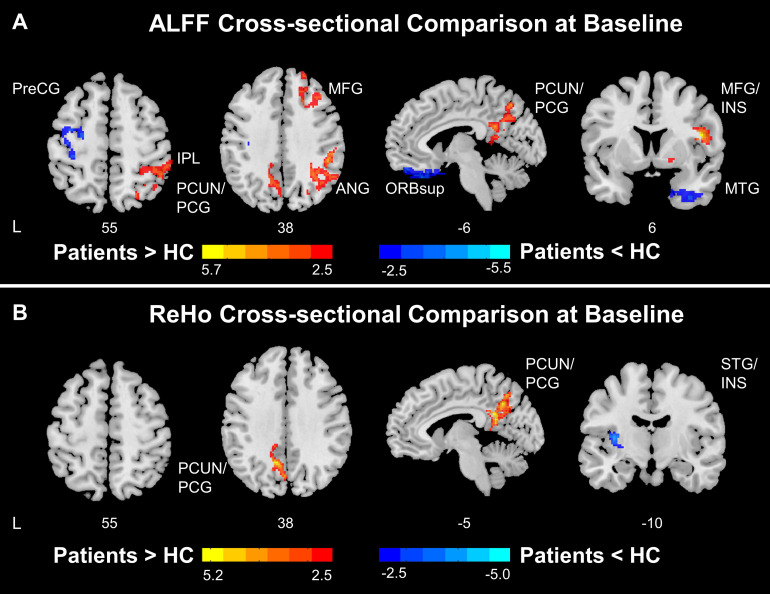
Functional disruptions in stroke patients. **(A)** ALFF comparison between HC and patients at baseline. At baseline, stroke patients showed reduced ALFF at ipsilesional SMN and increased ALFF at DMN and salience network comparing to healthy controls. **(B)** ReHo comparison between HC and patients at baseline. At baseline, stroke patients showed increased ReHo at DMN and decreased ReHo at insula comparing to healthy controls. Color bars indicate t-scores. L, Ipsilesional Hemisphere; DMN, Default Mode Network; SMN, Somatomotor Network; HC, Healthy Control; Precg, Precentral Gyrus; SMA, Supplementary Motor Area; IPL, Inferior Parietal; PCUN, Precuneus; PCG, Posterior Cingulate Gyrus; MFG, Middle Frontal Gyrus; ANG, Angular Gyrus; ORBsup, Superior Frontal Gyrus, Orbital Part; INS, Insula; MTG, Middle Temporal Gyrus.

Cross-sectional comparisons conducted at baseline indicated abnormal ALFF and ReHo patterns in stroke patients compared to healthy controls. Stroke patients showed lower ALFF in the ipsilesional precentral gyrus and postcentral gyrus and higher ALFF in the precuneus, posterior cingulate gyrus, angular gyrus, middle frontal gyrus, and contralesional insula (Figure 2A). Thus, these findings suggest that stroke patients had lower ALFF in ipsilesional SMN and higher ALFF in the DMN and contralesional SAN compared to healthy controls.

Stroke patients had higher ReHo in the precuneus and posterior cingulate gyrus and lower ReHo in the ipsilesional superior temporal gyrus and insula (Figure 2B). Collectively, the results indicate that stroke patients had higher ReHo in the DMN and lower ReHo in the ipsilesional SAN compared to healthy controls.

### Differential Changes in ALFF/ReHo in the MI-BCI and MI-BCI + tDCS Groups Post-intervention

**FIGURE 3:**
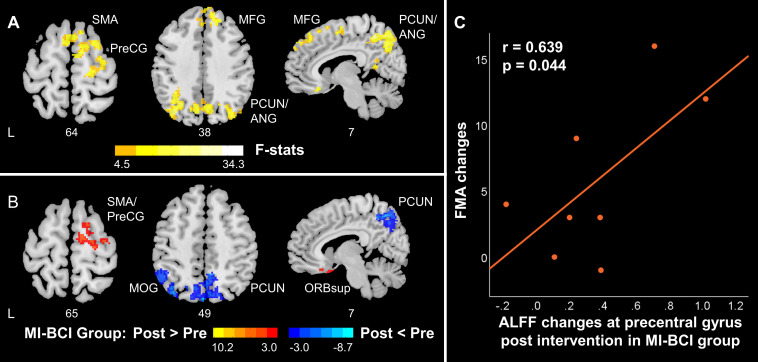
ALFF changes in contralesional SMN and posterior DMN in stroke patients along with motor function recovery. **(A)** The interaction between time and group. The MI-BCI group and MI-BCI + tDCS group showed divergent intervention effects at contralesional SMN and DMN. **(B)** Intervention effect in MI-BCI group. MI-BCI group showed increased ALFF at contralesional SMN and decreased ALFF at DMN. No such effect was observed in the MI-BCI + tDCS group. **(C)** Correlation between FMA changes and ALFF changes in the MI-BCI group. A positive correlation was observed between FMA changes and ALFF changes post intervention in the MI-BCI group at the precentral gyrus. No such correlation was observed in the MI-BCI + tDCS group. L, Ipsilesional Hemisphere; DMN, Default Mode Network; SMN, Somatomotor Network: HC, Healthy Control; Precg, Precentral Gyrus; SMA, Supplementary Motor Area; PCUN, Precuneus; PCG, Posterior Cingulate Gyrus; MFG, Middle Frontal Gyrus; ANG, Angular Gyrus; INS, Insula; MTG, Middle Temporal Gyrus.

**FIGURE 4:**
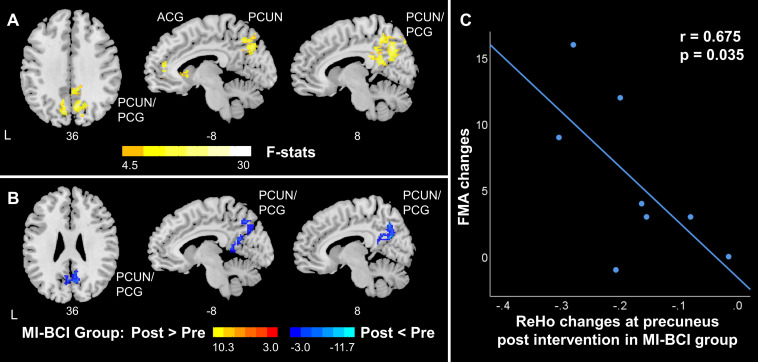
ReHo changes in DMN in stroke patients along with motor function recovery. **(A)** The interaction effect between group and time. The MI-BCI group and MI-BCI + tDCS group showed divergent intervention effects at DMN. **(B)** The intervention effect in the MI-BCI group. The MI-BCI group showed decreased ReHo at DMN. No such effect was observed in the MI-BCI + tDCS group. **(C)** Correlation between FMA changes and ReHo changes in MI-BCI group. A negative correlation was observed between FMA changes and ReHo changes post intervention in the MI-BCI group at precuneus. No such correlation was observed in the MI-BCI + tDCS group. L, Ipsilesional Hemisphere; DMN, Default Mode Network; PCUN, Precuneus; PCG, Posterior Cingulate Gyrus; ACG, Anterior cingulate and paracingulate gyri.

Significant interaction effects between intervention (pre-training, post-training) and group (MI-BCI group, MI-BCI + tDCS group) were found in ALFF and ReHo based on mixed-effect ANOVA, suggesting differential brain functional activity and connectivity changes in the MI-BCI group and MI-BCI + tDCS group. Specifically, differential ALFF patterns post-intervention were found in the contralesional regions within the DMN and SMN, including supplementary motor area, contralesional precentral gyrus, middle frontal gyrus, precuneus, and angular gyrus (Figure 3A). On the other hand, differential ReHo patterns were found in the precuneus and posterior cingulate gyrus (Figure 4A), suggesting that the MI-BCI group and MI-BCI + tDCS group exhibited differential ReHo patterns in the DMN post-intervention.

*Post hoc* analyses revealed that the MI-BCI group showed increased ALFF in the contralesional supplementary motor area and precentral gyrus post-intervention and decreased ALFF in the precuneus, inferior parietal lobule, and middle occipital gyrus (Figure 3B). The regions that showed changes post-intervention were very similar to regions that showed abnormal ALFF comparing to healthy controls at baseline (Figure 2A). Increased ALFF at baseline in the DMN was reduced post-intervention. Decreased ALFF in the ipsilesional SMN at baseline were not increased after training but increased ALFF was observed in the contralesional SMN. Similarly, decreased ReHo at bilateral precuneus and posterior cingulate gyrus was observed in the MI-BCI group post-intervention (Figure 4B). The altered regions were identical to the regions that showed abnormal ReHo changes in stroke patients at baseline comparing to healthy controls (Figure 2B). Higher ReHo in the DMN at baseline was reduced post-intervention.

In contrast, the MI-BCI with tDCS group had no significant changes in ALFF/ReHo post-intervention.

### Functional Connectivity Changes Between the SMN and DMN

**FIGURE 5:**
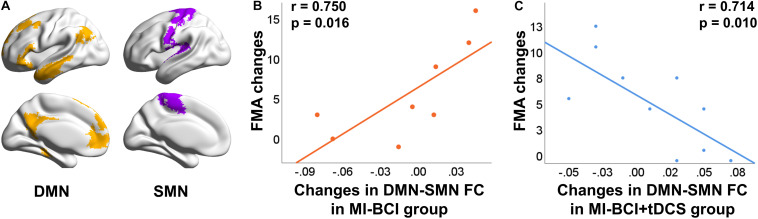
Differential correlation between FC changes and behavioral outcome changes using different intervention methods. **(A)** Stroke patients showed lower FC at SMN comparing to healthy controls at baseline (*p* = 0.030). **(B)** Correlation between FC changes and FMA changes in MI-BCI group. A positive correlation was observed between FMA changes and interconnectivity changes of DMN and SMN. **(C)** Correlation between FC changes and FMA changes in MI-BCI + tDCS group. A negative correlation was observed between FMA changes and interconnectivity changes of DMN and SMN.

Given the above ALFF/ReHo patterns mainly in the SMN and DMN, we then investigated if the FC in these two networks was disrupted in stroke patients and whether there was any intervention effect. As expected, stroke patients showed lower FC within the SMN at baseline compared to healthy controls (*p* = 0.03, effect size = 0.93) (Figure 5A) while no differences were found for FC within the DMN and FC between the SMN and DMN. Furthermore, no intervention effect (interaction or *post hoc* effects) was found.

### Association Between Brain Functional Measures and Motor Function Improvement

To find the association between changes in brain functional activity/connectivity and motor function post-intervention, we investigated the correlation between the ALFF/ReHo/FC changes and FMA changes.

A positive correlation (*r* = 0.639, *p* = 0.044) between ALFF changes in the precentral gyrus and FMA changes was observed in the MI-BCI group (Figure 3C), which indicated that patients who obtained higher ALFF increase post-intervention gained greater motor function improvement.

A negative correlation (*r* = −0.675, *p* = 0.035) between ReHo changes at precuneus and FMA changes was found in the MI-BCI group (Figure 4C). The association between decreased ReHo changes and increased FMA changes indicated that patients who had larger ReHo reduction gained greater motor function improvement.

Differential associations of the FC changes between SMN and DMN and FMA changes were observed in the MI-BCI group and MI-BCI + tDCS group. We found a positive correlation (*r* = 0.750, *p* = 0.016) between inter SMN-DMN FC changes and FMA changes in the MI-BCI group (Figure 5B) while a negative correlation (*r* = −0.714, *p* = 0.010) was reported in the MI-BCI + tDCS group (Figure 5C). The contradictory association in the two groups indicated that patients who obtained higher SMN-DMN FC increase would gain greater motor function improvement in the MI-BCI group while stroke patients in the MI-BCI + tDCS group would gain lower motor function improvement.

## Discussion

This study investigated the brain functional activity and connectivity changes in chronic subcortical stroke patients pre- and post-intervention with MI-BCI and tDCS. We found that stroke patients had lower ALFF in the ipsilesional SMN at baseline and had increased ALFF in the contralesional SMN in the MI-BCI group, providing new evidence for the compensation effect. Furthermore, the higher ALFF/ReHo in the DMN at baseline were decreased after MI-BCI training, illustrating the re-normalization of brain activity and connectivity post-intervention. More importantly, the divergent associations between SMN-DMN FC changes and FMA changes in the MI-BCI group and MI-BCI + tDCS group suggested differential mechanisms in the two approaches.

### Compensation Effect of the Contralesional SMN After MI-BCI

The ALFF findings suggest the possible beneficial effect of brain activity in the contralesional motor cortex after MI-BCI-based intervention. Stroke patients had lower ALFF in the ipsilesional SMN at baseline; and then the MI-BCI group showed increased ALFF in the contralesional SMN post-intervention. Importantly, increased regional brain activity in the contralesional motor cortex was positively associated with motor function improvement, suggesting a compensation effect of the contralesional SMN.

A predominant hypothesis of interhemispheric interaction is that the motor cortex of one hemisphere inhibits the contralateral homolog (Ferbert et al., 1992; Gerloff et al., 1998; Di Lazzaro et al., 1999; Liepert et al., 2000; Lotze et al., 2006; Dancause et al., 2015; Lüdemann-Podubecká et al., 2016). In line with this hypothesis, studies have reported persistent contralesional sensorimotor cortex hyperexcitability in patients post-stroke (Liepert et al., 2000; Bütefisch et al., 2003). Such hyperexcitability in the contralesional hemisphere might be harmful to recovery as it inhibits the excitability of the ipsilesional hemisphere (Loubinoux et al., 2003; Manganotti et al., 2008). Repetitive transcranial magnetic stimulation (rTMS) and tDCS have also been employed to inhibit the contralesional motor cortex to improve motor function recovery (Takeuchi et al., 2005; Nowak et al., 2008; Song et al., 2014; Matsuura et al., 2015; Lüdemann-Podubecká et al., 2016).

In parallel, recent BCI-related researches have shown contradictory results against the predominant hypothesis. Patients were reported to gain significant motor function improvement by utilizing the contralesional motor cortex in BCI therapy (Song et al., 2014; Young et al., 2014; Bundy et al., 2017). Although the role of the contralesional motor cortex is still unclear, evidence showed that the contralesional motor cortex excitability is positively correlated with recovery from stroke in chronic stroke patients (Lotze et al., 2006; Schaechter and Perdue, 2008; Young et al., 2016). One hypothesis on the function of the contralesional motor cortex is that it may provide additional neural resources for compensation of ipsilesional damages (Nowak et al., 2009; Butler et al., 2013; Touvykine et al., 2016). Our findings provide new evidence supporting the compensation effect of the contralesional SMN after MI-BCI stroke rehabilitation. Our previous study reported cortical activity changes measured by transcranial magnetic stimulation in the contralesional primary motor cortex were associated with functional improvement, also suggesting a possible role for the contralesional primary motor cortex in stroke recovery (Chew et al., 2020). Nevertheless, more effort should be made to study the role of the contralesional motor cortex in stroke rehabilitation, which could possibly depend on lesion size, stroke type, stroke stage, and BCI experiment design (Buetefisch, 2015; Dodd et al., 2017).

### Re-normalization of Brain Activity and Connectivity After MI-BCI

Stroke patients showed lower ALFF/FC in SMN and higher ALFF/ReHo in the DMN compared to healthy controls at baseline. Post-intervention, the MI-BCI group showed increased ALFF in the contralesional SMN and decreased ALFF/ReHo in the DMN. Our results match with previous findings on alterations of brain activity and connectivity in the DMN and the brain re-normalization hypothesis.

Alterations of brain activity and connectivity in the DMN were widely reported in stroke patients with impaired cognitive function (Tuladhar et al., 2013; Ding et al., 2014; Liu et al., 2014; Park et al., 2014; Wang J. et al., 2019). Stroke patients had significantly lower FC in the key DMN regions, namely precuneus and posterior cingulate gyrus compared to healthy controls (Tuladhar et al., 2013; Ding et al., 2014; Wang J. et al., 2019). Reduced ALFF and ReHo in the DMN were reported in stroke patients with early impairment in consciousness (Tsai et al., 2014). Our results showed higher ALFF and ReHo in the DMN in stroke patients at baseline compared to healthy controls. The difference might be due to different function impairments (cognitive impairment vs. motor function impairment) and different stroke stages (acute/subacute vs. chronic). A possible explanation for higher brain activity and connectivity in the DMN is the compensatory effect for motor function. More resources were required to achieve normal motor function due to the ipsilesional SMN decline, resulting in higher DMN activity in patients than healthy controls as compensation for declined SMN function (Pan et al., 2017).

More importantly, re-normalization of the brain activity and connectivity were observed post-intervention. The higher ALFF and ReHo in the precuneus and posterior cingulate gyrus at baseline reduced post-intervention in the MI-BCI group, which refers to re-normalization of the local activity and connectivity. Brain re-normalization in previous studies focused on alterations in FC. Increased FC after BCI rehabilitation therapy in the SMN and DMN where FC was lower at baseline was reported, which also hints at re-normalization of brain connectivity (Park et al., 2014; Tsuchimoto et al., 2019; Wang J. et al., 2019).

### Differential Mechanisms of MI-BCI and MI-BCI + tDCS

The efficacy of MI-BCI on improving motor function in stroke rehabilitation has been widely reported (Ang et al., 2009; Teo and Chew, 2014; Ang and Guan, 2015; Pichiorri et al., 2015; Ramos-Murguialday et al., 2019; Foong et al., 2020). Studies on tDCS also illustrated the beneficial effects of non-invasive cortical stimulation on stroke rehabilitation (Nitsche and Paulus, 2001; Zaghi et al., 2010; Madhavan et al., 2011; Allman et al., 2016; Elsner et al., 2018; Kashiwagi et al., 2018; Liu et al., 2018). However, the combination of MI-BCI and tDCS in this study did not further improve motor function than MI-BCI therapy without tDCS. Both the MI-BCI group and MI-BCI + tDCS group gained significant motor function improvement with no group differences reported regarding FMA scores. The differential patterns of brain activity and connectivity post-intervention and the divergent associations between these brain measures and FMA in the two groups underscored differential mechanisms underlying MI-BCI and tDCS. The MI-BCI group showed increased ALFF in the contralesional SMN and reduced ALFF/ReHo in the DMN post-intervention while no significant neuroimaging findings were observed in the MI-BCI + tDCS group. Furthermore, contrasting correlations were found between FC changes and clinical outcome changes in the MI-BCI group and MI-BCI + tDCS group. Our results indicated that MI-BCI and tDCS had potentially divergent ways of modulating functional brain activity. MI-BCI system enables stroke patients to move the impaired limb driven by a robotic arm via neural signals from the motor imagery of the arm and provides multisensory feedback to patients to enhance motor learning and recovery (Ang et al., 2014, 2009). tDCS modulates activity in both the motor and visual cortices and directly influences the excitability of the spinal cord (Nitsche and Paulus, 2001; Elsner et al., 2018). Generally, anodal stimulation increases cortical excitability, while cathodal stimulation decreases it. We suspect that the inhibition effect of cathodal might prevent or reverse the brain activity/connectivity changes in the MI-BCI + tDCS group, leading to insignificant results compared to MI-BCI only group and contradictory correlation with FMA scores. Consistent with our findings, another study reported that white matter integrity in the ipsilesional corticospinal tract and bilateral corpus callosum was increased but sensorimotor cerebral blood flow (CBF) was decreased, while in the MI-BCI group contradictory results or insignificant results were reported (Hong et al., 2017). Quantitative electroencephalographic (Q-EEG) features which changed significantly during these interventions were also mutually exclusive for MI-BCI and MI-BCI + tDCS group. The relative theta power was observed to be the signature monitory biomarker for BCI intervention whereas the tDCS group was characterized by a change in brain symmetry index, indicating distinct mechanisms of neuronal repair of the two approaches (Mane et al., 2019). The detailed explanation of changes in the brain requires further understanding of both MI-BCI and tDCS mechanisms. Our results suggest that a combined rehabilitation strategy should be designed considering not only the improvement of clinical outcomes but also changes in the brain.

### Limitations and Future Work

This is a pilot study of stroke rehabilitation involving complex multimodal design of neuroimaging, MI-BCI and tDCS, which is limited by the small sample size. Due to the limited sample, we were not able to control for stroke severity and lesion type or location in our analysis. Also, the possible inter-variability in conventional occupational therapy was not taken into account. Future research with large sample size should incorporate these potential important factors to evaluate the long-term rehabilitation effect. Another limitation is the lack of a tDCS-only group which would help to understand the mechanism of tDCS to facilitate the development of stroke rehabilitation strategies. Although the tDCS stimulation parameters applied in this study follow the standard protocol, it would be to study how stimulation parameters such as sites and placement of electrodes could impact the efficiency. Furthermore, while the current study focused on subcortical chronic stroke patients, it is crucial to design effective rehabilitations for stroke patients in acute or subacute phase moving forward.

## Conclusion

In conclusion, patients with chronic subcortical stroke showed lower ALFF/FC in the SMN and higher ALFF/ReHo in the DMN compared to controls at baseline. Such brain functional vulnerability patterns in the two networks was partially re-normalized post-intervention in the MI-BCI group while the MI-BCI + tDCS group showed no significant changes. Divergent associations between SMN-DMN FC changes and FMA changes were found in the MI-BCI group and MI-BCI + tDCS group. Our results suggested the potential differential or even opposite network-specific brain functional mechanisms underlying MI-BCI with and without tDCS in stroke recovery.

## Data Availability Statement

The datasets generated during and/or analyzed during the current study are available from the corresponding author upon reasonable request.

## Ethics Statement

The studies involving human participants were reviewed and approved by the National Healthcare Group Domain-Specific Review Board. The patients/participants provided their written informed consent to participate in this study.

## Author Contributions

MH codesigned the study with JZ, EC, and CG performed all the data analysis, and prepared the manuscript. H-JC, FJ, and JC assisted with data preprocessing and manuscript revision. JZ, CG, and XJ supervised and revised the manuscript. EC provided the data. ZL, WH, KA, KP, and H-JC assisted in data collection and preparation. All authors reviewed the manuscript.

## Conflict of Interest

The authors declare that the research was conducted in the absence of any commercial or financial relationships that could be construed as a potential conflict of interest.
