# Supplementary material for: Brain Functional Changes in Stroke Following Rehabilitation Using Brain-Computer Interface-Assisted Motor Imagery With and Without tDCS: A Pilot Study
Source: Front Hum Neurosci. 2021 Jul 16;15:692304. doi: 10.3389/fnhum.2021.692304 (PMC8322606; doi:10.3389/fnhum.2021.692304)
Supplement: Supplementary file 1 [file Data_Sheet_1.docx]

**Supplementary Figure 1. MI-BCI process.**


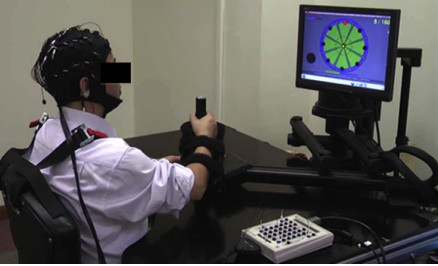


The MIT-Manus is a robot with 2 degrees of freedom that provides horizontal elbow and forearm reaching exercises using an 8-point clock-face drawing interactive video game. In this study, the stroke-affected upper limb of subjects from both groups was strapped to the Manus robotic exoskeleton. The subjects were instructed to imagine moving their stroke-affected hand toward the target indicated on the 8-point clock-face video game. They were also instructed to continue MI until successful or unsuccessful detection was indicated on the video screen. Voluntary movements during MI were restricted by locking the mobility of the Manus robot. If MI was successfully detected, visual and movement feedback was provided by the Manus robot through passive movement of the stroke-affected arm from the center toward the target displayed on the screen and back to the target along a predetermined robotic trajectory. This robotic movement forms a proprioceptive afferent feedback that closes the loop in providing a reward for performing MI.

Since not all stroke patients could operate EEG-based MI-BCI, the patients recruited in this study first underwent an MI-BCI screening session. In the screening session, a total of 160 trials of EEG that randomly comprised 80 MI conditions of the stroke- affected upper limb and 80 idle conditions were collected. The stroke patients’ abilities to operate MI-BCI were then evaluated based on the 10x10-fold cross-validations of the 160 trials of data collected using the Filter Bank Common Spatial Pattern (FBCSP) algorithm25 without any removal of artifacts such as the electro-oculogram. Subjects with MI-BCI classification accuracy >58% were then recruited for randomization.

The calibration session consisted of 4 runs of 40 trials each for a total of 160 trials, and an interrun break of at least 2 minutes was provided after each run. Each run randomly comprised 20 trials of MI of the stroke-affected upper limb and 20 trials of the idle condition. Each trial lasted approximately 12 seconds, and each run lasted approximately 8 minutes. The calibration session lasted approximately 1 hour inclusive of EEG setup time. A visual cue was used to prepare the subject, and subsequently another cue was used to randomly instruct the subject to perform either MI or the idle condition. The EEG segment of 0.5 to 2.5 seconds from the instruction cue was then extracted to train a subject-specific MI detection model using the FBCSP algorithm. No robotic feedback was provided in the calibration session.

The rehabilitation session comprised an evaluation portion and a therapy portion. The evaluation portion consisted of 40 trials that randomly comprised 20 trials of MI of the stroke-affected upper limb and 20 trials of the idle condition. Similar to the calibration session, a visual cue was used to prepare the subject, and subsequently an instruction cue was provided. The EEG segment of 0.5 to 4.5 seconds from the instruction cue was then extracted to classify the EEG segment to perform online detection of MI or the idle condition using the FBCSP algorithm. Once MI was detected, the robot was triggered to provide a feedback. The online accuracy of the evaluation portion of the rehabilitation session was then recorded based on the detection of MI or the idle condition compared with the instruction provided.

**Supplementary Figure 2. tDCS process.**


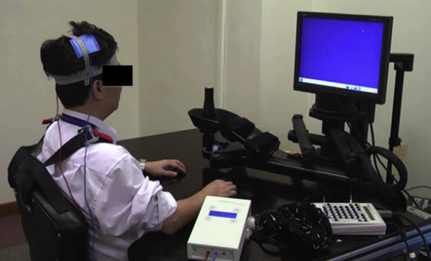


Direct current was delivered by a stimulator (NeuroConn, Germany) through rubber electrodes embedded in saline-soaked 50×70 mm2 sponge bags at an intensity of 1mA. The anodal electrode was placed over the ipsilesional M1 and the cathodal electrode was placed over the contralesional M1. Stimulation intensity was ramped up to 1mA over 30 s and maintained for 20min, before ramping down. Sham-tDCS was delivered by similarly ramping up to 1mA but maintained for only 20 s to give participants the same scalp sensation, before ramping down. tDCS intervention lasted for 20min for both groups so that participants were blinded to their group allocation. The low current density of 1 mA was chosen to prevent patient’s discomfort and allows application of tDCS for long periods of time.

**Supplementary Figure 3**. **Incidence maps of the lesions.**


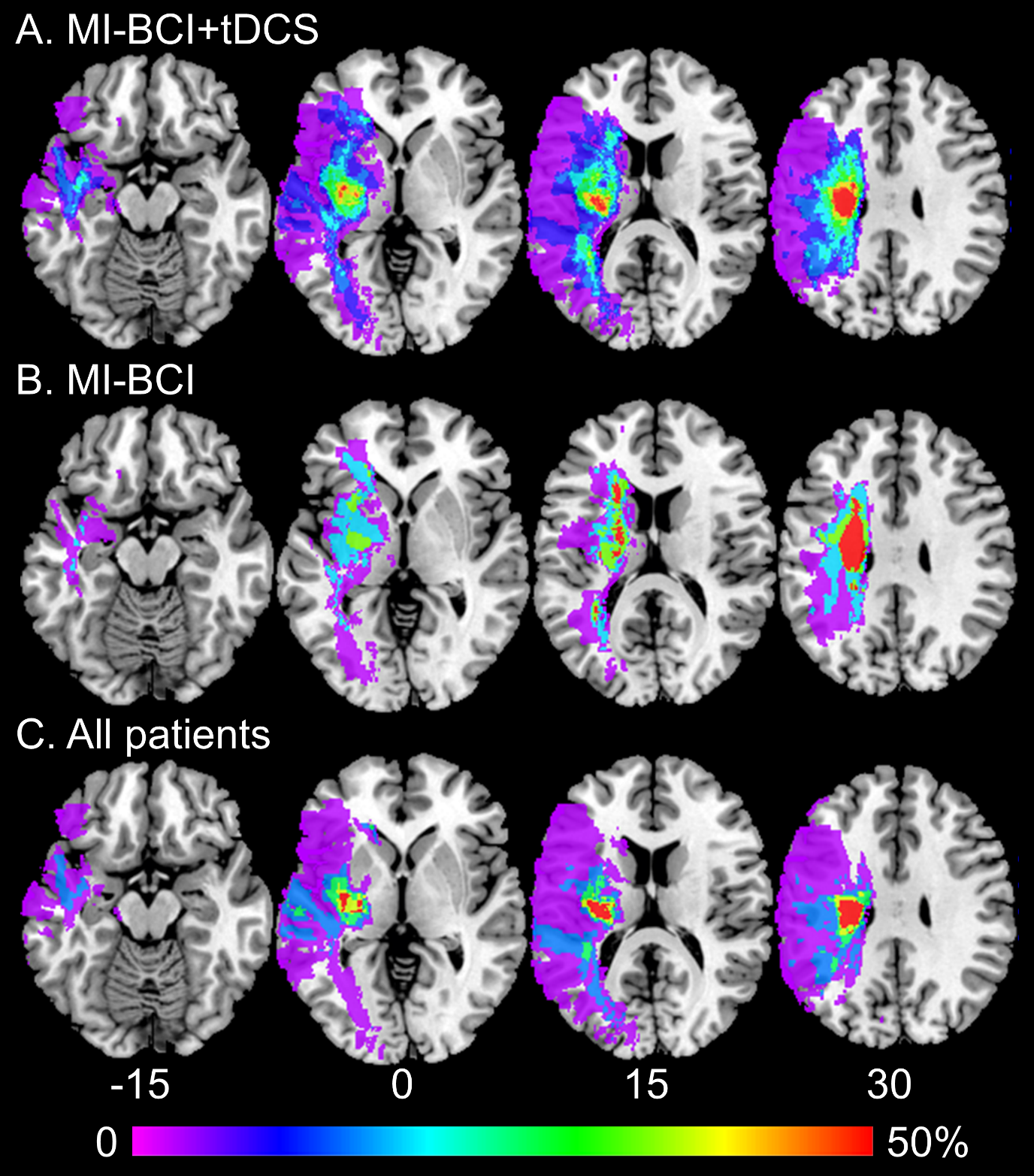


**Supplementary Table 1.** ROIs in the DMN and SMN.

| ROI names | X | Y | Z | ROI names | X | Y | Z |  |
| --- | --- | --- | --- | --- | --- | --- | --- | --- |
| **Left Hemisphere** | | | | **Right Hemisphere** | | | | |
| 'LH_SomMotA' | -14 | -38 | 74 | 'RH_SomMotA' | 4 | -38 | 62 |  |
| 'LH_SomMotB_Cent' | -62 | 0 | 20 | 'RH_SomMotB_Cent' | 64 | 2 | 18 |  |
| 'LH_SomMotB_S2' | -60 | -6 | 6 | 'RH_SomMotB_S2' | 62 | -6 | 10 |  |
| 'LH_SomMotB_Ins' | -32 | -24 | 4 | 'RH_SomMotB_Ins' | 36 | -24 | 2 |  |
| 'LH_SomMotB_Aud' | -54 | -10 | 4 | 'RH_SomMotB_Aud' | 56 | -8 | 0 |  |
|  |  |  |  |  |  |  |  |  |
| 'LH_DefaultA_IPL' | -48 | -76 | 34 | 'RH_DefaultA_Temp' | 62 | -8 | -16 |  |
| 'LH_DefaultA_PFCd' | -18 | 28 | 42 | 'RH_DefaultA_IPL' | 58 | -64 | 30 |  |
| 'LH_DefaultA_PCC' | -2 | -48 | 18 | 'RH_DefaultA_PFCd' | 28 | 38 | 42 |  |
| 'LH_DefaultA_PFCm' | -2 | 46 | -4 | 'RH_DefaultA_PCC' | 8 | -48 | 20 |  |
| 'LH_DefaultB_Temp' | -56 | -38 | -6 | 'RH_DefaultA_PFCm' | 4 | 52 | -6 |  |
| 'LH_DefaultB_IPL' | -58 | -52 | 26 | 'RH_DefaultB_Temp' | 64 | -34 | -2 |  |
| 'LH_DefaultB_PFCd' | -8 | 22 | 56 | 'RH_DefaultB_AntTemp' | 52 | 8 | -40 |  |
| 'LH_DefaultB_PFCl' | -42 | 12 | 52 | 'RH_DefaultB_PFCd' | 8 | 22 | 62 |  |
| 'LH_DefaultB_PFCv' | -44 | 36 | -12 | 'RH_DefaultB_PFCv' | 52 | 30 | -12 |  |
| 'LH_DefaultC_IPL' | -44 | -72 | 24 | 'RH_DefaultC_IPL' | 56 | -68 | 20 |  |
| 'LH_DefaultC_Rsp' | -12 | -60 | 12 | 'RH_DefaultC_Rsp' | 14 | -56 | 12 |  |
| 'LH_DefaultC_PHC' | -22 | -38 | -14 | 'RH_DefaultC_PHC' | 24 | -32 | -22 |  |

**Supplementary Table 2.** BCI performance.

| ID | Ave Accuracy | Std Accuracy | Ave Detection | Std Detection |
| --- | --- | --- | --- | --- |
| ***MI-BCI+tDCS Group*** | | | | |
| N001 | 75.05% | 14.97% | 94.82% | 3.61% |
| N005 | 56.75% | 8.08% | 86.13% | 18.90% |
| N006 | 70.25% | 11.39% | 71.94% | 15.62% |
| N010 | 60.50% | 11.71% | 87.38% | 10.14% |
| N015 | 80.75% | 13.59% | 80.13% | 16.40% |
| N025 | 54.75% | 13.41% | 88.75% | 13.13% |
| N027 | 49.50% | 10.33% | 74.04% | 17.45% |
| N029 | 50.25% | 0.79% | 100.00% | 0.00% |
| N035 | 65.25% | 12.88% | 83.94% | 14.48% |
| N037 | 65.75% | 6.02% | 69.00% | 15.36% |
| Mean | 62.88% | 10.32% | 83.61% | 12.51% |
| Mean STD | 10.45% | 4.29% | 9.97% | 6.18% |
| ***MI-BCI group*** | | | | |
| N007 | 53.75% | 5.68% | 90.06% | 10.27% |
| N009 | 79.25% | 10.34% | 87.19% | 9.59% |
| N011 | 56.75% | 8.82% | 88.55% | 8.31% |
| N019 | 53.50% | 3.94% | 73.18% | 23.36% |
| N021 | 52.75% | 4.32% | 91.06% | 12.99% |
| N031 | 55.25% | 6.61% | 94.56% | 8.79% |
| N030 | 57.00% | 6.95% | 91.52% | 8.17% |
| N032 | 50.75% | 7.36% | 81.06% | 16.46% |
| Mean | 57.38% | 6.76% | 87.15% | 12.24% |
| Mean STD | 9.08% | 2.16% | 6.89% | 5.30% |

**Supplementary Table 3a.** ALFF comparison at baseline.

| **Brain Regions** | **Hemisphere** | **Peak Coordinates** | **Cluster Size** | **Peak T Value** | **Effect Size**  **(mean±STD)** | **Peak Effect Size** |
| --- | --- | --- | --- | --- | --- | --- |
| **Patients > HC** |  |  |  |  |  |  |
| Precuneus/ Posterior cingulate gyrus | L | -10 -62 46 | 431 | 5.2418 | 0.0001±0.0065 | 1.1528 |
| Angular gyrus/ Inferior parietal lobule | R | 44 -66 30 | 1812 | 5.1099 | 0.0007±0.0122 | 0.8896 |
| Middle temporal gyrus | L | -66 -36 -6 | 304 | 4.3894 | 0.0001±0.0074 | 0.7788 |
| Middle frontal gyrus/ Superior frontal gyrus/ Insula | R | 44 6 18 | 2276 | 5.6201 | 0.0008±0.0127 | 1.1250 |
| **Patients < HC** |  |  |  |  |  |  |
| Precental gyrus/ Postcentral gyrus | L | -32 -8 48 | 726 | -4.3356 | 0.0002±0.0069 | 0.6928 |
| Middle temporal pole | R | 18 10 -32 | 389 | -3.7417 | 0.0001±0.0063 | 0.5734 |
| Superior frontal gyrus, orbital part | R | 22 34 -24 | 898 | -4.3785 | 0.0003±0.0099 | 0.8028 |
| Superior temporal gyrus | L | -48 -36 20 | 510 | -5.6178 | 0.0003±0.0102 | 1.0946 |
| Fusiform gyrus | R | 34 -68 -12 | 300 | -4.8585 | 0.0001±0.0055 | 0.9216 |

**Supplementary Table 3b.** ReHo comparison at baseline.

| **Brain Regions** | **Hemisphere** | **Peak Coordinates** | **Cluster Size** | **Peak T Value** | **Effect Size**  **(mean±STD)** | **Peak Effect Size** |
| --- | --- | --- | --- | --- | --- | --- |
| **Patients > HC** |  |  |  |  |  |  |
| Precuneus/ Posterior cingulate gyrus | L | -8 -48 26 | 652 | 5.1805 | 0.0003±0.0091 | 0.9917 |
| Cerebellum | R | 14 -92 -26 | 309 | 4.6987 | 0.0001±0.0050 | 0.6645 |
| Cerebellum | L | -42 -64 -26 | 223 | 4.3796 | 0.0001±0.0048 | 1.0063 |
| **Patients < HC** |  |  |  |  |  |  |
| Superior temporal gyrus/insula | L | -38 -32 10 | 921 | -4.6678 | 0.0004±0.0125 | 0.9354 |
| Calcarine sulcus | R | 24 -58 6 | 331 | -4.1559 | 0.0001±0.0057 | 0.5597 |

**Supplementary Table 4a.** ALFF interaction effect.

| **Brain Regions** | **Hemisphere** | **Peak Coordinates** | **Cluster Size** | **Peak F Value** | **Effect Size**  **(mean±STD)** | **Peak Effect Size** |
| --- | --- | --- | --- | --- | --- | --- |
| Supplementary motor area | R | -2 0 58 | 652 | 21.3561 | 0.0003±0.0103 | 1.3348 |
| Precentral gyrus | R | 24 -24 74 | 280 | 12.4052 | 0.0001±0.0060 | 0.7753 |
| Middle frontal gyrus | R | 14 36 58 | 735 | 14.9206 | 0.0003±0.0098 | 0.9325 |
| Middle occipital gyrus | R | 40 -74 24 | 371 | 22.9306 | 0.0002±0.0087 | 1.4332 |
| Precuneus/ Angular gyrus | L&R | 8 -50 52 | 3914 | 34.2711 | 0.0022±0.0271 | 2.1419 |
| Inferior temporal gyrus | L | -44 -24 -30 | 345 | 20.5512 | 0.0002±0.0099 | 1.2844 |
| Cerebellum | L | -16 -48 -48 | 269 | 30.7413 | 0.0001±0.0082 | 1.9213 |
| Gyrus rectus | L | 4 28 -22 | 265 | 12.6230 | 0.0001±0.0077 | 0.7889 |

**Supplementary Table 4b.** ALFF intervention effect.

| **Brain Regions** | **Hemisphere** | **Peak Coordinates** | **Cluster Size** | **Peak T Value** | **Effect Size**  **(mean±STD)** | **Peak Effect Size** |
| --- | --- | --- | --- | --- | --- | --- |
| **MI-BCI Group** |  |  |  |  |  |  |
| **Post > Pre** |  |  |  |  |  |  |
| Supplementary motor area/ Precentral gyrus | R | 20 6 58 | 534 | 7.3404 | 0.0012±0.0462 | 7.6973 |
| Superior frontal gyrus | L | 14 -2 -28 | 674 | 6.1248 | 0.0015±0.0494 | 5.2590 |
| Inferior temporal gyrus | L | -48 -12 -44 | 239 | 4.9617 | 0.0005±0.0266 | 3.5169 |
| Cerebellum | L | -24 -56 -50 | 183 | 10.1847 | 0.0005±0.0432 | 14.8184 |
| **Post < Pre** |  |  |  |  |  |  |
| Precuneus/ Inferior parietal lobule | R&L | -2 -78 52 | 2889 | -8.5867 | 0.0065±0.0966 | 10.5329 |
| Middle occipital gyrus | R | 36 -72 30 | 185 | -7.1480 | 0.0004±0.0295 | 7.2990 |
| **MI-BCI+tDCS Group** |  |  |  |  |  |  |
| None |  |  |  |  |  |  |

**Supplementary Table 5a**. ReHo interaction effect.

| **Brain Regions** | **Hemisphere** | **Peak Coordinates** | **Cluster Size** | **Peak F Value** | **Effect Size**  **(mean±STD)** | **Peak Effect Size** |
| --- | --- | --- | --- | --- | --- | --- |
| Precuneus/ Posterior cingulate gyrus | R&L | 8 -62 40 | 1199 | 33.061 | 0.0006±0.0157 | 2.0663 |
| Anterior cingulate gyrus | L | -4 42 2 | 306 | 15.5411 | 0.0001±0.0073 | 0.9713 |

**Supplementary Table 5b**. ReHo intervention effect.

| **Brain Regions** | **Hemisphere** | **Peak Coordinates** | **Cluster Size** | **Peak T Value** | **Effect Size**  **(mean±STD)** | **Peak Effect Size** |
| --- | --- | --- | --- | --- | --- | --- |
| **MI-BCI Group** |  |  |  |  |  |  |
| **Post < Pre** |  |  |  |  |  |  |
| Precuneus/ Posterior cingulate gyrus | R&L | 8 -64 26 | 590 | -11.163 | 0.0016±0.0621 | 17.8108 |
| **MI-BCI+tDCS Group** |  |  |  |  |  |  |
| None |  |  |  |  |  |  |
